# The Comic History of British Nursing

**Published:** 1913-03-08

**Authors:** 


### The Comic History of British Nursing.

A History of Nursing. Edited, and in part written, by Lavinia Dock, Secretary of the International Council of Nurses. Vols. III. and IV. (G. P. Putnam's Sons. 1912. Price 21s. net.)

To compile a history of nursing which shall be even moderately complete and adequate is an ambitious undertaking. For nursing is no longer one of those professions which exist only in Great Britain, the United States, and Germany. It flourishes and spreads in many countries, where until lately good nursing was non-existent, and therefore unappreciated. It follows that any full history of modern nursing must be the work of many writers, and that the editorial function is one of much difficulty and labour. Miss Dock very courageously accepts entire responsibility for the work of her contributors, even "for the interpretation or colour of the narrative and for personal touches." This frank admission is vastly to the credit of her loyalty, but very little to that of her discretion; for it is precisely the abundance and the quality of such interpretations, colours, and personal touches which deprives her volumes of all claim to any historical value whatever.

The third volume of the series (the first of the two now published) deals with many matters which will interest nursing and hospital circles in this country. For it takes up first of all the story of the past thirty years or so of nursing in Great Britain, to which 115 pages are allotted. After that the growth of nursing in the United States, in the countries of Northern Europe, and in France is described; but to Great Britain more than one-third of the whole volume is devoted. Now bitter controversies, as we all know, have stirred the British nursing world during the period with which Miss Dock's third volume deals; and the embers of them are still inclined to smoulder, though the fierce blaze is long overpast. The protagonists, or most of them, in those controversies are still alive—a fact which by no means lightens the task of passing judgment impartially upon the merits of the disputes and differences of opinion to which we have referred. Still, if history is to be written at all, or is to be worth writing, it must be critical, judicial even; and it certainly ought to be unbiassed and impartial.

That, however, is not Miss Dock's way, we are sorry to say. Her idea of compiling history is to allow a small clique, consisting of those who most actively sustained one particular side of the controversies in question, to collaborate in mutual laudation and in depreciation of all opponents. Their own propaganda is described in terms of the most extravagant encomium; the views of all others are dismissed without argument as the outcome of sheer malice and wrongheadedness. Everything which is done by Miss Dock's friends is described (by themselves, be it noted) as the result of "dogged tenacity," "intrepid daring," "unerring intuition," "divination," and so forth. Their own photographs and those of their supporters are sprinkled freely through the letterpress; in short, history as they write it is resolved into a mutual admiration society on a grand scale.

It is easy to understand that it is impossible to take seriously this farrago of prejudice masquerading as history. The misrepresentations of fact alone are sufficient to condemn it; and when to these the "interpretations and personal touches" are added, the narrative does indeed become full of "colour." The climax is reached when the name of Mrs. Bedford Fenwick is placed on a level with that of Florence Nightingale! (p. 32).

The fourth volume is concerned with the progress of nursing during modern times in Germany, Switzerland, Holland, Belgium, in the East, and in various colonies, islands, and foreign countries. There is much that is of great interest and permanent value in the record; and the volume as a whole shows what Miss Dock might have accomplished in the third volume also, if only she had felt the responsibility of writing history as something different from an opportunity to advertise a propaganda and to fawn upon its chief exponents.

Miss Dock is the authoress of more than one good book, and the first two volumes of the "History of Nursing," in which she had the invaluable and powerful aid of Miss Adelaide Nutting, who is so wise a bibliophile as to give her a literary force and status few women possess. We mention all this to prove what good and impartial work Miss Dock is capable of producing.

Volumes I. and II. of this "History of Nursing" are history, and will always have a permanent value as such, especially when used in conjunction with "Hospitals and Asylums of the World." They deal with the evolution of nursing systems from the earliest times, and the foundations of the first English and American training schools for nurses, and as such have possessed a permanent value.

These last two volumes, for the reasons we have given, have failed altogether to attempt, much less to reach, the high standard of the first two volumes, and we would suggest to the publishers that, if a second edition be ever called for, it would be a service to literature to produce the first two volumes as a separate book of permanent importance and historical value. If they should ever republish at their discretion the third and fourth volumes, they should appear as a separate book altogether, and be classified in their catalogue amongst works of comic humour.

A farrago of prejudice masquerading as history can only have a very transitory and passing interest, and it would have been juster to the best work of which she is capable, and to Miss Adelaide Nutting, if Miss Dock had published what she now denominates Volumes III. and IV. as a separate work, having no connection whatever with the first two volumes, which are entitled to rank and to be preserved as an admirable epitome of universal nursing history.

