# A Comic History of British Nursing

**Published:** 1913-06-14

**Authors:** 


### ERRATA.

"A Comic History of British Nursing."

It has been pointed out that an error has crept into the last two paragraphs of the review under this heading in our issue of March 8, 1913, page 622, and the following should be substituted for them:—

The third of the last two volumes, for the reasons we have given, has failed altogether to attempt, much less to reach, the high standard of the first two volumes, and we would suggest to the publishers that if a second edition be called for it would be a service to literature to produce the first two and the last volume as a separate book of permanent importance and historical value. If they should ever republish at their discretion the third volume it should appear as a separate book altogether, and be classified in their catalogue amongst works of comic humour.

A farrago of prejudice masquerading as history can only have a very transitory and passing history, and it would have been juster to the best work of which she is capable, and to Miss Adelaide Nutting, if Miss Dock had published what she now denominates Volume III. as a separate work, having no connection whatever with the first two and last volumes, which are entitled to rank and be preserved as an admirable epitome of universal nursing history.

